# In-Session Reflective Functioning: Relationship With the Presence and Depth of Work on Conflict or Personality Functioning

**DOI:** 10.3389/fpsyg.2021.725739

**Published:** 2021-09-20

**Authors:** Cecilia de la Cerda, Paula Dagnino

**Affiliations:** ^1^Faculty of Social Science, Department of Mediations and Subjectivities, Universidad de Playa Ancha, Valparaíso, Chile; ^2^Millennium Institute for Research on Depression and Personality, Santiago, Chile; ^3^Center for the Research on Psychotherapy – CIPSI, Santiago, Chile; ^4^Faculty of Psychology, Universidad San Sebastian, Santiago, Chile

**Keywords:** single case, therapeutic focus, reflective functioning, psychotherapy process, episodes of change

## Abstract

Mentalizing, conceived as the capacity to attribute intentional mental states as implicit or underlying behavior of an individual or others, has gained interest within psychodynamic clinical research due to its potential as a change mechanism. Variations and qualities of mentalization have been studied through reflective functioning (RF). But only few studies are analyzing it throughout the psychotherapeutic interaction, identifying its level for therapists and patients. In contrast, brief psychodynamic therapy has a long tradition for establishing a focus to be worked upon. Lately, a multischematic focus has arisen, considering both conflict and personality functioning focuses as key elements on successful psychotherapies. This study aimed to identify mentalizing manifestations of patients and therapists through change episodes of one successful brief psychodynamic therapy and establish the relationship between these mentalizing manifestations and the type and depth of the therapeutic focus being worked on (conflict or personality functioning). Only 37.5% of speaking turns were able to be coded with RF; 77% of these had moderate to high RF and 22% had low or failure RF. The patient had 91% of low or failure RF, while the therapist only had 9% of low or failure RF. As for moderate to high RF, patients had 39%, while therapists had 61%. The patient showed a similar number of low or failure RF interventions and moderate to high RF interventions in conflict episodes. Meanwhile, the therapist only performs moderate to high-level RF interventions. In episodes in which personality functioning is worked on, both patient and therapist show a greater presence of interventions of moderate to high levels of RF. Finally, mentalizing interactions and non-mentalizing interactions were found on segments with conflict, and only mentalizing interactions were found on personality functioning segments.

## Introduction

Authors from psychoanalysis (e.g., Luborsky, 1984; Green, 1975; Horowitz et al., 1993a,b; Green, 1996) postulate that the clinical approach on the psychodynamics of patients must be understood based on two different central thematics (therapeutic focus): intrapsychic conflicts and malfunctions or deficits in functioning (Killingmo, 1989, 1990; Schüßler, 2004; Dreher, 2005, 2006; Sugarman, 2006). Moreover, any work on the psychodynamic process leads to a better understanding of the contents of the mind. Consequently, enhancing mentalizing is an essential mechanism through which psychotherapy works (Choi-Kain and Gunderson, 2008) and becomes a key concept for understanding change. It is then clinically relevant to identify the interventions of the therapist that improve the ability of the patient to reflect and become aware of the relationship between their own mental states and behaviors. Particularly, to identify them in those segments in which conflict or personality functioning is being worked on, expecting that there will be differences between them in the mentalization quality of the participants.

In the first place, authors situate the concept of mentalization within the psychodynamic tradition, in what they call “a doubly configured epistemic space,” the empirical perspective of developmental theorists with the clinical understanding of psychoanalytic theorists (Fonagy, 1994). The term was initially derived from the philosophical tradition of authors such as Dennet (1978, 1983), Wittgenstein (1953, 1969), and Davidson (1983) and is then taken up in the field of psychology by theorists of mind and metacognition, who explore the way it develops in the first years of life (Baron-Cohen et al., 1985). Later, Fonagy et al. (1991) attempted to demonstrate that infant understanding of mental worlds of an individual and others has profound dynamic implications for the organization of the self (Fonagy et al., 1991). As an antecedent of what will later be defined as reflective function, Fonagy et al. (1991) found the distinction between a *pre-reflective self* , referring to an immediate or unmediated experience of life and a *reflective self* or reflective function of the self that operates as an internal observer of mental life.

On the contrary, the therapeutic focus has also had an extensive theoretical development in the history of psychoanalysis (e.g., Malan, 1963; Sifneos, 1979). It can be considered the center around which psychotherapy is organized and a change mechanism itself (Balint et al., 1972; DeLaCour, 1986; Poch and Maestre, 1994; Scaturo, 2002). The identifying focus needs an initial dynamic formulation. Therefore, the focus is considered ideographic to each patient. One system that allows for the identification of focus is the operationalized psychodynamic diagnosis (OPD Taskforce, 2008), which considers focus as different specific areas that are significant for the psychodynamics of the patient and must be worked on throughout the process (Grande et al., 2004). Three focuses can be established with this system: dysfunctional relational pattern, conflict focus, and personality functioning focus. Works on conflict and personality functioning are relevant to this study since they are the most psychodynamic ones.

Conflict focus alludes to the work on those unconscious coalitions between motivational groups that leads to an elevated internal state of tension. It assumes that human behavior is constantly influenced by unconscious desires, thoughts, and representations. In OPD, conflict refers to a rigid pattern of experiences that, in certain situations, lead to the same pattern of behavior without the person being aware of it or being able to change it voluntarily (OPD Taskforce, 2008). The work on conflict is the closest to depth psychology, which aims at revealing unconscious conflicts, that is, making the patient aware of them and returning them to their responsibility are strongly oriented toward the psychoanalytic treatment technique, in the form of clarifications, confrontations and interpretations (Rudolf, 2002).

Personality functioning focus alludes to the observable manifestations of structural conditions (the actual use of capabilities; Dahlbender et al., 2006). It evolves around two lifelong tasks, the development of capacities for interpersonal relatedness and self-definition or identity, underpinned by functions oriented toward self-regulation and the relationship between the self and its internal and external objects. Focus on this area must look at those major vulnerabilities of the patient (see for more detail OPD Taskforce, 2008). Personality functioning as a goal of psychotherapeutic interventions is not systematically discussed in the literature. In Wöller (2001), supportive and interactional techniques for building these functions are mentioned. These interventions have also been proposed as a way to reinforce these same functions (Kernberg, 1999). Working on these vulnerabilities means fundamentally accepting and trying to accept the patient in the way they experience and act. This means that therapists must first respond to the concerns, questions, doubts and expectations of the patient and be more active, supportive, and affirming than, for example, during the work on conflict (Rudolf, 2002). Finally, the studies of Karlsson and Kermott (2006) and Katznelson (2014) suggest that brief therapies using supportive techniques would not promote changes in RF.

Process research on this subject (Mentzos, 1991; Dagnino, 2021) has shown that some segments of psychotherapy revealed a prevalence of the work on conflict focus, others on personality functioning, being almost complementary throughout the process. For relational pattern, its presence is almost stable during the process, considering it as an epiphenomenon. This complementarity was also seen during psychodynamic processes, finding that work on conflict occurs mainly at the beginning of the process, while work on personality functioning occurs toward the end of the process (Dagnino, 2012, 2021). Moreover, it is expected that during the work on conflict and personality functioning through the process, mentalization may be improved since these are the core themes for psychodynamic work. There is a need to understand how reflective functioning is enhanced on a micro perspective level and understand how the interaction between patient and therapist promotes this ability when conflict or personality functioning is being worked on.

Considering a form of social cognition, mentalizing has been defined as an imaginative mental activity (Bateman and Fonagy, 2012, 2019). It refers to the capacity to understand other people and oneself in terms of intentional states of minds (Fonagy et al., 2012), awareness of mental states, which includes “perceiving and interpreting the feelings, thoughts, beliefs, and wishes that explain what people do” (Bateman and Fonagy, 2019, p. 3). It is acquired in early childhood and is closely associated with attachment and affective regulation systems. Its presence and its failures or dysfunctions are the basis of some etiological models of mental disease (Linehan, 1993; Clarkin et al., 2006; Fonagy et al., 2015). There is extensive evidence that shows that patients suffering from severe mental disorders to a greater or lesser degree display failures in their mentalizing skills. Thus, and in general terms, psychoanalysis promotes mentalization (Sugarman, 2006). Successful psychotherapies positively influence these skills (Levy et al., 2006a,b; Fonagy and Luyten, 2009). As a significant component of therapeutic action, variations and qualities of mentalization have been measured within the context of psychotherapy, confirming its role as a relevant factor of change (Bateman and Fonagy, 2006, 2012; Clarkin and Levy, 2006; Levy et al., 2006a,b; Luyten et al., 2015a,b; de la Cerda et al., 2019).

Furthermore, in close agreement with the findings of psychotherapy research, it has been advanced that mentalizing is a specific relational skill (Luyten et al., 2012), which is expressed through mentalizing in action, that is, the interactional and dynamic process in which each participant remains attentive to his/her mental states while at the same time being aware of the mind of other person (Allen and Fonagy, 2006). From this perspective, mentalizing can be thought of as a specific aspect of general regulation focused on a particular object: the mental states of the self and the other. It has been suggested that it recursively performs a double function: it is an emergent phenomenon of regulation with the other and at the same time contributes to or hinders the regulatory system, depending on its operational quality (de la Cerda, 2017; de la Cerda et al., 2019). From this perspective, it is possible to envision the function acquired by mentalizing in processes of reciprocal influence and self-regulation, such as the psychotherapeutic interaction.

Studies conducted in the psychotherapeutic process reported two types of interactions between patient and therapist, *mentalizing interactions* and *non-mentalizing interactions* (de la Cerda et al., 2015; de la Cerda, 2017; de la Cerda et al., 2019). A mentalizing interaction is observed when the patient increased their reflective capacity, which could be attributed to the interventions made by the therapist. On the contrary, a non-mentalizing interaction refers to when the patient decreases the quality of their reflective functioning.

Both the OPD approach and the mentalization approach postulate that psychoanalytic therapy centered on interpretation is the most appropriate tool for the work of unveiling unconscious conflicts (Fonagy et al., 1993; Rudolf, 2004). On the contrary, when facing deficits in functioning, the proposed work approach should be different, not centered on interpretation, but taking as objects those deficits and disturbances more actively (Bateman and Fonagy, 2004, 2006; Rudolf, 2004, 2007, 2010).

Only few studies were found that review the relation of mentalizing and conflict/personality functioning focus, and they were mainly on its relation with personality functioning. Müller et al. (2006) stated that the conceptualizations of both RF and personality functioning (OPD) are based on the assumption that the self is actualized in the process of developing relationships and, therefore, in the psychotherapeutic process. They can be understood as both pointing to structural diagnosis and rating psychic functions. However, they have different validity claims, RF from the framework of attachment theory and OPD personality functioning as integrating different psychodynamic theories as a product of clinical psychoanalytic research.

The approach of identifying RF during the psychotherapeutic process is a novel approach. The goal of this study, therefore, was to identify RF of both therapist and patient and evaluate them on segments where conflict or personality functioning is being worked on. The design was exploratory with the analysis of a single case. The aims were (1) to examine the quality of RF for both patient and therapist, (2) to find out whether there are differences on RF of patient and therapist during conflict/personality functioning segments and (3) to identify mentalizing and non-mentalizing interactions on both types of segments. Our hypothesis with respect to the objectives has several points: (1) we expect that there would be differences between patient and therapist on RF as previous research has shown; (2) we expect that on conflict segments, RF of the patient will be high since the work is on making the patient aware of unconscious conflicts; on the contrary, it is expected that RF will be low on personality functioning patients because the work is on those major vulnerabilities, through support interventions which lead to more regulation; and (3) with respect to mentalizing and non-mentalizing interactions, it is expected that on segments where personality is worked on, non-mentalizing interactions will be present, since support techniques are mainly used, which does not lead to insight.

## Materials and Methods

### Design and Participants

This is an exploratory study with a qualitative approach. The study was based on a cross-sectional single case design: Qualitatively identifying conflict or personality functioning and the reflective function coding of each speaking turn of therapist and patients. Quantitative analyses were made through the scoring of each variable.

The case was selected from a group of therapies recorded on the postdoctorate research of one of the authors (PD). The pool of therapies was broad from individual to group therapy and from psychodynamic to cognitive-behavioral, each of them with different lengths. This case was chosen because it was the only psychodynamic-focused psychotherapy case. The need to choose a psychodynamic therapy case was related to the dimensions being studied, that is, mentalization and therapeutic focus. Both concepts come from a psychoanalytic perspective. Finally, this was a successful therapy, concluding from a self-report instrument evaluating well-being. This is a questionnaire that was answered by the patient before and after the process (Outcome Questionnaire, OQ-45.2, Lambert et al., 1996; Von Bergen and de la Parra, 2000).

As part of the project, both therapist and patient consents were obtained to use the material in subsequent research by the responsible researcher, and they were contacted again later to confirm approval. Authorization for using these data for this study was granted by the Ethics Committee from Universidad Alberto Hurtado, following the declaration of Helsinki (64th WMA General Assembly, Fortaleza, Brazil, October 2013).

The psychodynamic therapy selected consisted of 21 sessions. The material for this study was already segmented in change episodes due to the previous project, which were the ones analyzed here. Change episodes are those segments in session in which there is an intensification of the process of change, culminating in a specific change moment (identified from a list of generic change indicators, see Krause et al., 2007). From the 21 sessions, 24 episodes of change were analyzed.

All of these 24 episodes were codified with the Focus Presence and Depth Scale (FPDS, Dagnino and de la Parra, 2010), which is specified in the instrument section. For each of the episodes, the dysfunctional relational pattern, conflict focus, and personality functioning focus were identified. Relational pattern is a focus that has been shown to be the expression of conflict and personality (Grande, 2007); therefore, this study selected only those episodes that showed a high presence of conflict or personality functioning focuses (Dagnino, 2012). This leads to only nine episodes, of which four had a predominance on conflict focus and five had the predominance of work on personality functioning.

### Participants

The therapy was conducted in Chile by a male psychiatrist and psychoanalyst with 25 years of experience. The patient was a woman who attended an outpatient psychotherapy unit at a university clinic. More details on the patient will be given later.

### Instruments

#### Operationalized Psychodynamic Diagnosis (OPD-2, OPD Taskforce, 2001)

Operationalized psychodynamic diagnosis is a diagnosis system that proposes an articulated integration of fundamental dimensions for a global comprehension of a patient. It consists of five axes; three of them are psychodynamic, which are evaluated for this study: Axis 2: interpersonal relationships, Axis 3: conflict, and Axis 4: personality functioning. Its scoring includes a training and clinical application manual and response forms for each axis for an easier and more reliable application. The rating for this system has received considerable empirical support (Cierpka et al., 2001; Zimmermann et al., 2012; Dinger et al., 2013).

As for each axis, a focus can be identified through the evaluation of its particular dynamics. Specifically, of interest for this study, conflict focus can be selected from seven types of conflict: (1) Individuation vs. dependency, (2) Submission vs. control, (3) Desire for protection and care vs. autarky (self-sufficiency), (4) Self-worth conflict, (5) Guilt conflict, (6) Oedipal sexual conflict, and (7) Identity conflict. On the contrary, personality functioning focus can be selected from eight personality domains, (1) self-perception (2) object perception, (3) self-regulation, (4) regulation of relationships, (5) internal communication, (6) external communication, (7) attachment to internal objects and (8) attachment to external objects.

#### Foci Presence and Depth Scale (Dagnino and de la Parra, 2010)

It allows measuring the degree of presence and depth level of a focus, in a given segment of psychotherapy sessions. FPDS consists of determining the specific formulation and focus for the patient. With this information, the presence and depth of each focus can be scored on a 3 points scale: 1: vague reference, 2: knowledge and exploration of focus, or 3: work on focus. To identify in which episode conflict or personality functioning was prevalent, only level 3 was considered. For example, if one episode shows level 3 on conflict and personality functioning level 1 or 2, it will be regarded as an episode with a prevalence of conflict.

#### Reflective Functioning Scale (Fonagy et al., 1998)

Reflective Functioning Scale (RFS) enables the assessment of mentalizing operationalized as reflective functioning (RF). The scale was designed to respond to the Adult Attachment Interview (AAI; Main et al., 2003), making it possible to identify textual passages of reflective functioning, categorize them, and evaluate their quality. Examples of RF are coded on an 11-point scale from−1 (failure or anti-reflective) to 9 (exceptionally reflective). Fonagy et al. (1998) distinguished between two main levels, negative (−1 to 2) to low (3–4) vs. average (5–6) to high (7–9) RF (Taubner et al., 2012).

Some studies have applied RFS in other contexts. Relevant for this study is its use in transcriptions of psychotherapeutic sessions (Karlsson and Kermott, 2006; Szecsody, 2008; particularly, de la Cerda, 2017; de la Cerda et al., 2019). Two trained raters reviewed the transcriptions, identifying passages of RF in the therapist and the patient and assessing their quality. It is worth mentioning that these studies reported the existence of turns of speech that cannot be coded with the RFS. These have been called *non-passages* [between 50 and 70% of speech turns, de la Cerda (2017)].

### Procedures and Data Analysis

The sessions were video and audio recorded and later transcribed for their analysis. Each of the procedures described below was independently conducted by trained raters.

#### Determination of Relevant Episodes

For this study, the episodes of change were already identified and delimited. This was the material on which the analyses were performed. Although it was not part of the procedure for this study (only an input), it is worth detailing the procedure because of its relevance. Episodes of change are special segments of the therapeutic session (Elliot, 1984; Timulak, 2007) that make it possible to understand the connection between the therapeutic exchange and its outcome. For this study, change episodes (Krause et al., 2006) were analyzed. A change episode is considered as the segment of the patient–therapist interaction where a moment of change occurs. For the identification of change episodes, raters must identify a moment of change (through a list of Generic Change Indicators, Krause et al., 2006). This moment signals the end of the episode. Its beginning is established retrospectively by identifying the moment at which the participants start to talk about the content of the change (Krause and Dagnino, 2005). The episodes used in this study were analyzed by other research groups, having two pairs of trained coders who analyzed all the videos and transcripts of the sessions. Their codings were validated through intersubjective agreements (see Flick, 2004).

#### About the Identification of Focus

Two trained raters received the videos and transcripts of the two first sessions. With the use of the OPD manual, they developed a psychodynamic formulation identifying the particular foci (dysfunctional relational pattern, conflict, and personality functioning) for the patient. The interrater reliability kappas ranged from 0.45 to 0.76, which can be considered good (Fleiss, 1981; Cicchetti, 1994). Later, raters received the episodes of change and had to identify the presence and depth of each focus with the FPDS. This was done through intersubjective consensus.

#### About the Identification of Type and Quality of RF

The type and quality of RF of each participant during the episode were codified with the RFS (Fonagy et al., 1998). For the nine episodes, a total of 272 speaking turns were analyzed with RFS, using AtlasTi (7.0). The scale was applied to the transcripts of the sessions, identifying low or failure RF and moderate to high RF. To identify passages, two raters trained in the use of the RFS coded the transcripts of the episodes. For the assignment of a quality score to the passages, raters follow the scoring guidelines included in the RFS (Fonagy et al., 1998), ranging from −1 to 9 and record speaking turns during which each exemplar took place. The former makes it possible to differentiate between low or failure RF passages and moderate to high RF passages.

## Results

### Case

Patient A was a 42-year-old woman, married, with four children (all in school). She studied economics, and her actual occupation was as head of the sales department of a small factory of furniture. Her husband was the owner and general manager of the company, therefore her boss. She complained that she was not happy at work and in general in all her interpersonal relationships. Besides, she referred that this happens especially at work, mainly because of her decision-making difficulties.

Her physical posture is hunched, with his hands together giving the impression of a submissive attitude. Her psychomotor skills are restricted due to inhibition in her expression. She seems to be of a younger age, sometimes behaving like a frightened little girl. In her history, she refers to having suffered mistreatment (physical and psychological) by her husband, and 1 year ago, she discovered his infidelity, which led her to a suicide attempt. She then began pharmacological treatment, which she stopped because her husband threw away the medication. She defines herself as “crazy,” a hard worker who sometimes does not know if she is doing things right and finds it difficult to make decisions.

She defines her husband as authoritarian, who gives her orders both at home and at work.

She is the eldest of four siblings. Her father was abusive with his siblings but not with her, so she stayed “quiet.” Her mother had states of madness (which appear to be psychotic) during the childhood of the patient, with many confusing moments for her.

### General Results

From the 272 speaking turns, only 102 (37.5%) were considered as passages able to be coded with RFS. The rest, 170 (62.5%), were non-passages turns of speech.

Considering the passages identified by the RFS, regardless of the participant, on the 102 turns of speech coded with RF, corresponding to the nine episodes, it was found that 79 turns (77.45%) belonged to passages of moderate to high RF, 23 turns (22.55%) to passages of low or failure of the RF (see Table 1).

**Table 1:** Frequency of data.

**Session**	**Episode**	**Total of speaking turns in the episode**	**Total of speaking turns coded with RFS**	**Patient**		**Therapist**		
				**Number of speaking turns with low or failure on RF**	**Number of speaking turns with moderate to high RF**	**Number of speaking turns with low or failure on RF**	**Number of speaking turns with moderate to high RF**	**Thematic focus worked on the episode**
3	Epi1	23	11	5	1	1	4	Conflict
4	Epi2	15	8	0	4	0	4	Personality
4	Epi4	11	5	0	1	0	4	Conflict
5	Epi6	113	35	8	10	0	17	Conflict
10	Epi13	25	12	1	5	1	5	Conflict
14	Epi17	9	7	5	0	0	2	Conflict
14	Epi18	59	18	1	8	0	9	Personality
21	Epi23	12	4	1	1	0	2	Personality
21	Epi24	5	2	0	1	0	1	Personality
	Total	272	102	21	31	2	48	

**Figure 1:**
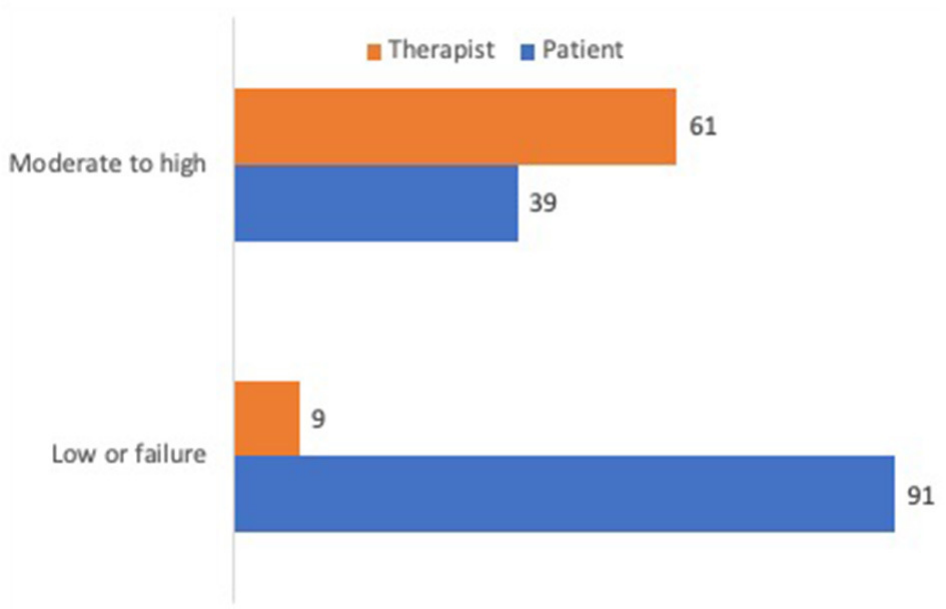
Percentage of RF for patient and therapist.

When observing the difference between patient and therapist of the specimens coded by the scale, it was found that the patient had 91% of low or failure on RF, while the therapist only had 9%. As for moderate to high RF, the patient had 39%, while the therapist had 61% (see Figure 1).

### Mentalization of Patient and Therapist During Episodes of Conflict or Personality Functioning Focus

Table 2 shows, in the number of turns of speech, the frequency of statements in which patient or therapist is showing a low or failure, or moderate to high RF quality. It can be observed how in those episodes in which conflict work is being done, the patient presents slightly more low or failure RF, while the therapist shows a high frequency of moderate to high RF. On the contrary, when working on personality functioning, both patient and therapist show a high amount of moderate to high interventions compared with low or failure RF.

**Table 2:** Frequency of low or failure and moderate to high RF in each participant on conflict or personality functioning focus episodes.

	**Patient**		**Therapist**	
	**Low or failure RF**	**Moderate to high RF**	**Low or failure RF**	**Moderate to high RF**
Conflict focus episode	19	17	2	32
Personality functioning focus episode	2	14	0	16
Total	21	31	2	48

### Mentalizing Trajectories Through the Episodes

**Figure 2:**
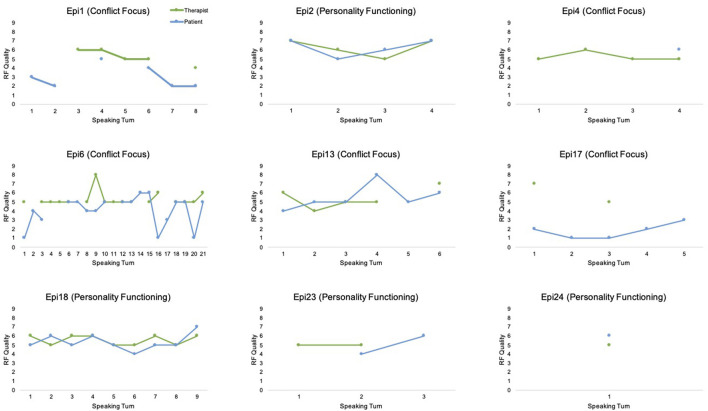
Quality of mentalization of therapist and patient in each episode.

From a descriptive point of view, Figure 2 represents fluctuations in the quality of mentalization (−1 to 9) of therapist and patient in each of the episodes analyzed. It is interesting to observe the different trajectories of the episodes. It is possible to visually identify a harmonized or synchronous therapeutic work between patient and therapist, as in episode 2 and episode 18, with similar qualities of RF for both participants. Other interactions suggest miscoordination, as in episode 17, in which the therapist presents moderate and high RF exemplars, but the patient remains at low or failing RF scores; or episode 6, with moments of marked differences between the reflective functioning of therapist and patient. In the following section, we will make a more detailed analysis of the characteristics of these interactions.

### Mentalization Interactions and Non-mentalizing Interactions in Episodes of Conflict or Personality Functioning Focus

Based on Figure 2, we will describe the results of the interweaving of the therapeutic work between patient and therapist in those episodes that predominate a focus on conflict or personality. As mentioned in the introduction, previous studies have identified two types of patient–therapist interactions, namely, mentalizing and non-mentalizing interactions (de la Cerda, 2017), depending on whether, due to the therapeutic work, the patient increases or decreases his or her reflective functioning.

Mentalizing interactions are characterized by the patient starting with low or failure mentalization, increasing in quality during the course of the interaction, presumably due to the interventions of the therapist, and ending the interaction with the patient mentalizing normally (moderate to high). On the contrary, non-mentalizing interactions usually begin with the patient in low or failure, or with a moderate to high mentalization, but in them the characteristic is that the interaction ends with the patient in low or failure, either because the therapist cannot help them to raise their mentalization quality or because, as a result of the interaction with the therapist, the patient stops mentalizing.

In this study, it was found that in those episodes in which conflict is predominantly worked on, both mentalizing and non-mentalizing interactions appear. On the contrary, when working predominantly on personality functioning, only mentalizing interactions appear.

In order to account for these results (qualitative and exploratory), example segments of these types of interaction in different types of episodes will be presented. Thus, a segment that exemplifies a non-mentalizing interaction and another with a mentalizing interaction in episodes where conflict is at work will be presented below. Then we will proceed to show the mentalizing interactions that occur in the episodes with an example where personality is worked on.

### Non-mentalizing Interaction in Conflict Episodes

**Figure 3:**
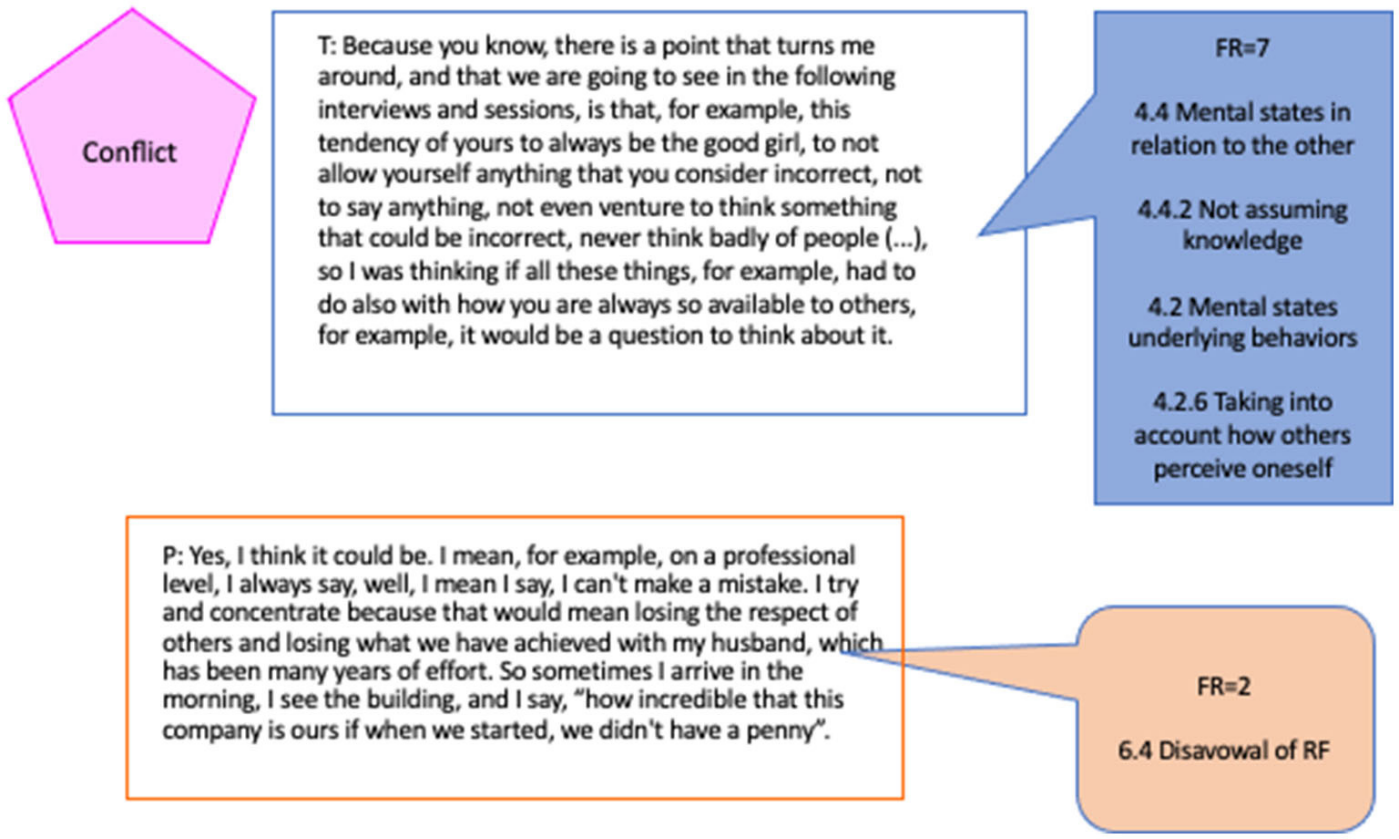
Non-mentalizing interaction in conflict (Epi1).

This is a typical example of a non-mentalizing interaction that can be attributed to a misalignment between the RF quality of patient and therapist. As can be seen in the content (see Figure 3), the therapist is working on the submission conflict. In an intervention with a high reflective level (RF = 7), the therapist presents an initial hypothesis about this conflict. However, perhaps precisely because of the initial mismatch between the interpretation offered and the reflective level with which the patient initiates the therapy, she fails, giving a concrete answer, externalizing and avoiding going deeper. The therapist asks for an affective and psychological meaning. The patient refers to concrete aspects of her working life, thematically disengaging from the invitation of the therapist to mentalize.

Figure 3 shows, in addition to the RF quality, the categories that, according to the RFS, characterize the coded passages (see RFS, Fonagy et al., 1998, p. 14 and following).

### Mentalizing Interaction in Conflict Episode

In the following example, we will see the other type of mentalizing interaction that was prevalent in this type of episode. In this case, we will see the function that mentalization acquires in interventions in which the therapist accompanies the patient in a more contingent and close way. From the perspective of OPD, the episode begins by referring to the relationship between the two prevalent conflicts selected as focus (submission/control and care vs. autarchy). The therapist (T) shows the patient (P) how having a more active, less submissive attitude activates the care conflict, the fear of not being loved or even punished. “T: (.) you say ‘I make decisions, but then comes the fear of having done something wrong? or of being punished?' or, or, or, that someone will get angry with you as in this case your husband?” In terms of reflective functioning, this is a demand question. By definition, it is a question that probes for RF (Taubner et al., 2012), those that demand from the other a demonstration of their capacity for reflective-self function (Fonagy et al., 1998). In terms of RF, this is a demand question. It is also a question, precisely about the relationship between mental states and behaviors, between making decisions, feeling fear, and to what that fear might be attributed. Regarding its quality, it shows a moderate RF (FR = 5) on the part of the therapist.

Two interventions from the patient follow this demand question: first, she responds from a failure of the reflective function (FR = 1): “P: well, in fact, he always gets angry if I don't go to work.” A one-dimensional representation of her own and the mental states of the other, which is a failure since, directly asked about the relationship between the anger of her husband and her fear, she generalizes, externalizes, and simplifies the reference with a superficial attribution.

The therapist tries to deepen this line of analysis, “T: but before you told me that you had made a decision and then you had started to doubt, (.) so now we see that it seems that this doubt has to do with this.” The therapist centers the dialogue around the relationship between the indecision of the patient and this reflection about protecting a child. Again, it is an example of moderate RF, a simple to understand one, in that he paraphrases what the patient herself points out and only links back to what she had given a hint that it might be related.

The intervention invites the patient to go deeper: “P: (.) then I said ‘no if I tell him something, he won't say anything to me, he will stay calm, but then will take it out on my son when he gets there, and he will scold him, he will insult him', so I said ‘yeah, I better not tell him anything', and I didn't tell him anything, I just ignored him.” This part of the response has a pre-reflective functioning (FR = 4); the patient refers to what she thinks and how that reflection leads her not to say anything to her husband. It is pre-reflective because, although she does not make the complete and transparent relationship between her mental states and the question of the therapist, it is noticeable that she can look at herself and analyze what thoughts motivated her action. Besides differentiating herself from the interpretative proposal of the therapist, she does not accept that it is fear of her husband but rather a strategy to protect her son.

The therapist orders the idea and paraphrases it: “T: That is, if you do something he doesn't like, then he punishes you; there is a retaliation about that.” With a better-quality response (FR = 5), the patient can think about what motivates the aggressive behavior of her her husband, why he prefers her to be at work, and what he does when she does not obey him: “P: Yes, maybe, I have thought so, because I know that he was very upset that I did not go to work, maybe it makes him feel safer that I am at work. I have sometimes thought that it is a punishment system that he uses. It is like: if I don't go, he is grumpy the rest of the day.”

### Mentalizing Interaction in Personality Functioning Focus Episodes

We have chosen the following two examples because we consider them to express the function that mentalization has acquired in episodes focusing on personality functioning, which, moreover, belong to the final sessions of therapy. They are both mentalizing interactions since these were the prevalent interactions in these episodes.

Episode 23 is a segment where personality functioning predominates specifically on the dimension of regulation with others. The work on the capacity for asking for help is one of the dimensions of personality functioning. This is the penultimate episode which is in the last session of the process. The therapist remembers how difficult it was for her to accept that she needed help. Also, he shows the patient how difficult it can be to say goodbye and finish this process, and how this is important since it sets a boundary and a compromise that both agreed. He reinforces the resources that the patient has. The patient says “I never talked about anything with anyone, so maybe these are experiences that also tell me that maybe I will have to work more on my social side, because there may not always be a specialist to tell things to, but maybe I could, at some point, find a person I could trust and perhaps that would also help me, I think, but that's like a project that is far away from me” (RF = 6).

**Figure 4:**
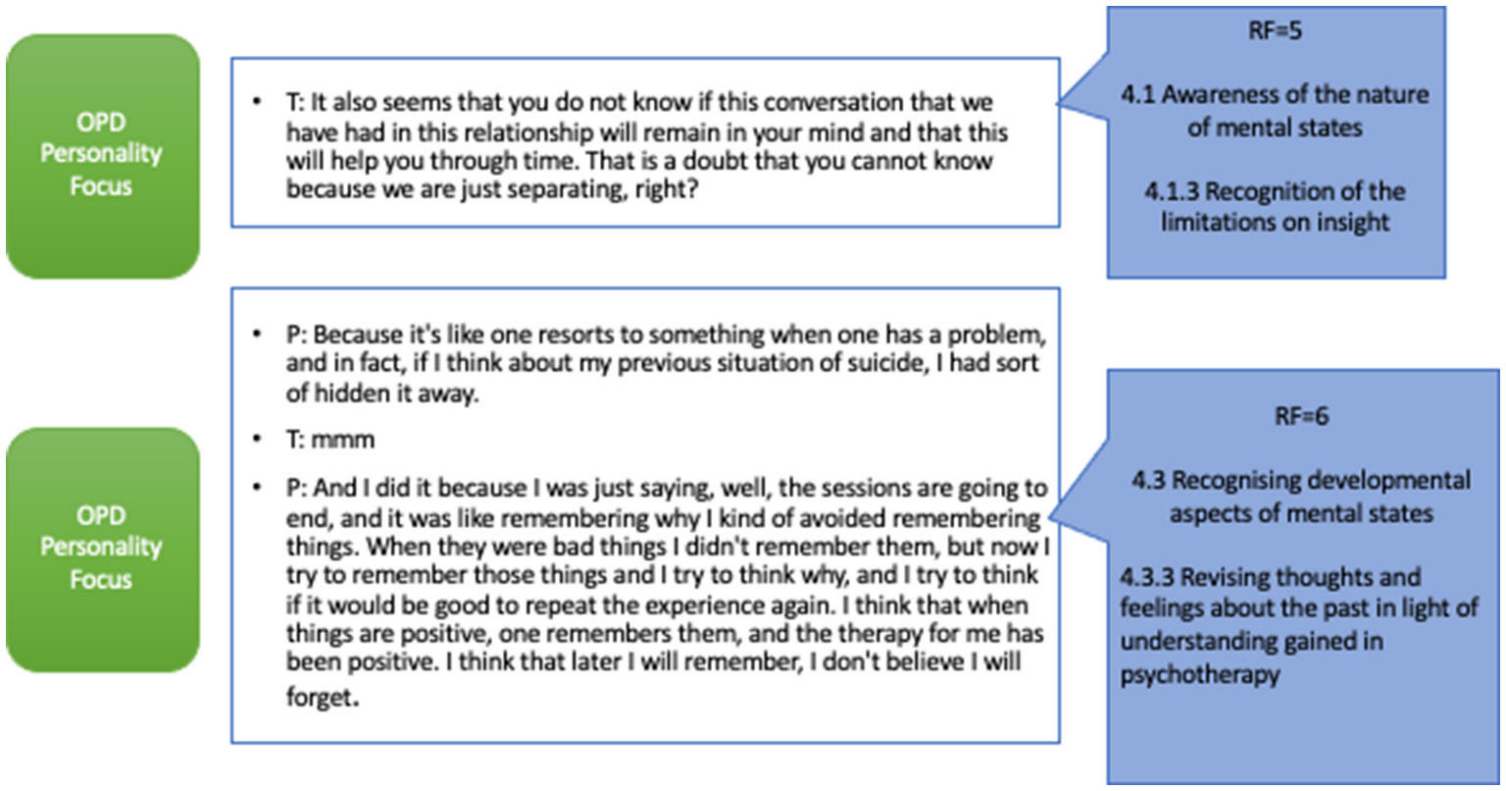
Mentalizing interaction in personality focus (Epi24).

Finally, in Figure 4, corresponding to the last episode (epi24), the therapist is working predominantly on personality functioning, in this case, in the area of self-worth, seeking to evaluate the capacity of the patient to incorporate positive introjects, allowing another characteristic of the mentalizing work to manifest itself during the work on the structure/personality. The therapist uses a level of RF, close to the statements of the patient both in the reflective quality and in the terminology used. The patient can then take up this invitation, developing the idea, alluding to the evolutionary aspects of mental states, reviewing her previous moderate feelings in the light of the understanding she now has of herself, giving an account, in passing, of the processes of change she experienced during therapy.

## Discussion

This study sought to explore the mentalizing manifestations of both patient and therapist in those segments where the therapeutic focus of conflict or personality functioning predominates and to identify mentalizing and non-mentalizing interactions in each of these segments.

In the analysis of reflective functioning, more than half of the speaking turns were non-passages, which coincides with what has been found in other studies (e.g., de la Cerda, 2017, de la Cerda et al., 2019). Previous studies have shown that dissimilarities are observed between the passages/non-passages ratio in different therapies, with a higher proportion of passages in patients with difficulties in interpersonal functioning (de la Cerda, 2017). Keeping in mind the particular and functional characteristic of explicit mentalizing noted by Fonagy and Luyten (2009), it can be argued that an excess of explicit mentalizing activity, as generally detected by RFS, is indicative of a greater need for regulation, that is, a hyper-mentalizing mode of functioning and probably concomitant with an arousal activation in one or both participants of the interaction (Fonagy et al., 2011). If this were the case, the increased mentalizing activity would be necessary as a regulatory mechanism in the face of the emotional intensity of the psychotherapeutic relational encounter (de la Cerda, 2017).

Considering the level of RF regardless of the participants, it was found that there is a more significant presence of turns of speech with moderate to high RF in general terms. This may be related to the fact that the segments analyzed are episodes of change (Krause et al., 2006, 2007), virtuous moments in therapy that shape a subjective understanding of oneself that is different from the previous one. They culminate in constructing a subjective psychological theory based on biography, which can be considered the final product of therapeutic change (Krause and Dagnino, 2005). Hence, episodes of change are unique segments of the therapeutic dialogue and sought-after scenes that define the purpose of psychotherapy and test its efficacy and meaning in terms of a logic of results. In segments of change of the patients, it can be expected a high mentalization.

Globally, when analyzing the different types of episodes (conflict/personality functioning), it was observed that the therapist mainly performs medium or high-level RF interventions in those episodes where conflict is predominantly worked. We know that for therapists trained in the psychoanalytic line, working on conflict configurations is a central element of any treatment (Smith, 2003), so it is understood that the therapist performs interventions of a higher reflective level. Psychoanalytic tradition has emphasized work on conflict as a sign of success, and therefore, processes are considered successful only if patients show insight into these themes (Kiesler, 1983). For the patient, during these episodes, she shows the same amount of responses of low or failure RF or moderate to high RF, which could be showing the mobility in the work that is required. Concerning the episodes in which work predominates over personality functioning, both therapist and patient showed medium to high levels of RF. The episodes of personality functioning focus occur in the last sessions of the process, as was found on another study (Dagnino, 2021). This may indicate that there may be a consolidation or synchrony in the level of work between both participants.

Finally, the mentalizing interactions during the episodes showed that on those episodes where the work is predominant in the conflict configurations, mentalizing and non-mentalizing interactions were found. The non-mentalizing interactions occurred mainly when the therapist made interventions with a high RF level, which caused a failure in the mentalization of the patient. This may be understood as the work on conflict implies techniques such as clarification, confrontations and interpretations (Rudolf, 2002), and it can be experienced as intense, painful, and with high emotional arousal by the patient, leading to failure. It becomes apparent that when the therapist performs interventions of similar levels to the patient, the patient increases her RF level from moderate to high. Perhaps, the therapist must perform less complex interventions to achieve better mentalization in the patient on those themes that elicit high emotions. As Kiesler (1983) suggests, pressure should be applied to the conflicts, but gently initially, to intensify the therapeutic work later on.

Contrary to what we expected when the patient and therapist are working on personality vulnerabilities, no failures on RF were observed from the patient. In these segments, any therapist intervention of low or failure RF or medium to high RF level generated a response in the patient of a medium to high RF level. As work on vulnerabilities in personality functioning can be equated with work on the aspects of personality disorders, it was to be expected that mentalizing would be disrupted and prementalizing modes would appear (Bateman and Fonagy, 2019). However, a hypothetical way to understand this different than expected outcome is to consider that interventions aimed at improving or working on those vulnerabilities are mainly directive, clarifying interventions that are in the support role (Luborsky, 1984; Rockland, 1989), and therefore, they produce a decrease in distress and emotional intensity and a tendency to greater regulation. All of the above would correlate with higher RF scores.

Several limitations must be taken into account. Because it is an exploratory study of just one case, we cannot determine causal relationships. Even though analyzing a single case allows for a deeper comprehension of the phenomena, expanding to include other patients with different baseline levels of functioning would help broaden the conclusions of this study.

Another aspect that could also not be reviewed due to the length of the case analyzed was the differences at different moments of therapy. In a previous study by one of the authors (de la Cerda, 2017), related to a 3-year therapy, the differences between three phases in the therapy—initial, middle, and final—were analyzed based on episodes of change. The observations indicated that the therapist maintains a relatively stable level of RF while the patient increases it considerably toward the end of the process. It would be of great interest to analyze in other studies the impact that this type of interaction has on the different phases of therapy, depending on whether conflict or personality is worked on from the perspective of the OPD.

Additionally, the analysis was made only on change episodes of the psychotherapeutic process. It would be interesting to consider different segments of the process to compare the interaction between therapist and patient on their mentalizing manifestations.

We hope that this study provides a valuable springboard to further research and a better comprehension of brief psychodynamic therapy.

## Data Availability Statement

The datasets presented in this article are not readily available because confidentiality. Requests to access the datasets should be directed to Paula Dagnino, pauladagnino@gmail.com.

## Ethics Statement

The studies involving human participants were reviewed and approved by Ethics Committee from Universidad Alberto Hurtado. The patients/participants provided their written informed consent to participate in this study.

## Author Contributions

All authors contributed to the data analysis and the writing of the manuscript and approved the submitted version.

## Funding

This study was supported by the Universidad de Playa Ancha de Ciencias de la Educación, Universidad Alberto Hurtado, Universidad San Sebastian and Innovation Fund for Competitiveness (FIC) from the Ministry of Economy, Development and Tourism, the ANID Millennium Science Initiative/Millennium Institute for Research on Depression and Personality-MIDAP ICS13_005.

## Conflict of Interest

The authors declare that the research was conducted in the absence of any commercial or financial relationships that could be construed as a potential conflict of interest.

## Publisher's Note

All claims expressed in this article are solely those of the authors and do not necessarily represent those of their affiliated organizations, or those of the publisher, the editors and the reviewers. Any product that may be evaluated in this article, or claim that may be made by its manufacturer, is not guaranteed or endorsed by the publisher.
